# Investigation of eco-friendly fluorescence quenching probes for assessment of acemetacin using silver nanoparticles and acriflavine reagent

**DOI:** 10.1038/s41598-023-31106-9

**Published:** 2023-03-14

**Authors:** Rana Ghonim, Mohamed I. El-Awady, Manar M. Tolba, Fawzia A. Ibrahim

**Affiliations:** 1 Department of Pharmaceutical Analytical Chemistry, Faculty of Pharmacy, Mansoura University, Mansoura, 35516 Egypt; 2 Department of Pharmaceutical Chemistry, Faculty of Pharmacy, Delta University for Science and Technology, International Coastal Road, Gamasa, 11152 Egypt

**Keywords:** Drug discovery, Chemistry, Nanoscience and technology

## Abstract

The non-steroidal anti-inflammatory medication acemetacin was assessed via two straightforward green spectrofluorimetric techniques. The quenching-dependent derivatizing spectrofluorimetric reactions are the master point of this study. Acriflavine-based method (Method I) depends on forming an ion association complex between acriflavine and the drug in a ratio of 1:1, decreasing the former's fluorescence intensity. Acriflavine or Ag NP's intensity-related quenching action goes linearly with the acemetacin concentration in the 2.0–20.0 µg/mL and 1.0–16.0 µg/mL ranges, respectively. The second quenching mechanism depends on using the silver nanoparticles (Ag NP's) as a fluorescence probe (Method II); Ag NP's were prepared from reducing silver nitrate using sodium borohydride. Both methods could be applied to determine pure and pharmaceutical dosage forms of acemetacin. The methods proved valid according to the international conference on harmonization (ICH) guidelines. In addition to this, this work has been estimated under green criteria assessment tools. There is no significant difference between the proposed and the comparison methods after the statistical interpretation.

## Introduction

**Figure 1:**
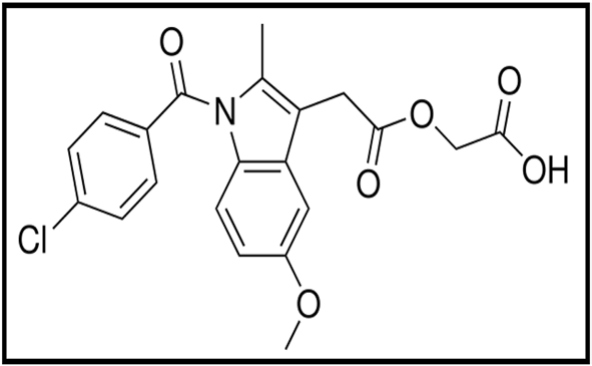
The structural formula of acemetacin (ACM).

Acemetacin (ACM) is O-[(1-p-Chlorobenzoyl-5-methoxy-2-methylindol-3-yl) acetyl]glycolic acid ester of indomethacin^1^ (Fig. 1). It is also listed in British Pharmacopoeia (BP)^1^ and United States Pharmacopoeia (USP)^2^. ACM is (a prostaglandin-endoperoxide synthase inhibitor) a non-steroidal anti-inflammatory drug. Acemetacin is eliminated by both hepatic and renal routes, although pharmacokinetics is not affected by moderate renal or hepatic impairment and appears to be unchanged in the elderly^3^. In clinical trials, acemetacin exhibits better gastric tolerability than its active metabolite indomethacin. Its pharmacological activity is due to acemetacin and its major metabolite, indomethacin. Usual oral doses are 120 to 180 mg daily in divided doses.

(Derivative spectrophotometry, liquid chromatography^4^), (stability-indicating Spectrophotometry, HPTLC^5^), and electrochemical techniques^6^ are variable analytical methods for ACM determination. No spectrofluorometric approaches have been published for ACM determination.

Acriflavine is an acridine fluorescence dye; chemically is 3,6-diamino-10-methylacridin-10-ium chloride. It served as a fluorescent and absorbent probe for spotting nucleic acid structures. Antimalarial, interferon-like activity^7^, antiviral^8^, antimicrobial activity^9^ anti-cancer activity, AIDS treatment, anti-bacterial and local antiseptic activity are its pharmacological actions. Due to its spectroscopic and pharmacological characteristics could be employed as a diagnostic tool and an effective laser dye^10^. Some drugs could be easily determined through their quenching of acriflavine, so their determination could be easily calculated through the difference in fluorescence intensity. Ascorbic acid^11^, ketoprofen, and ibuprofen^12^, olsalazine and sulfasalazine^13^ and furosemide^14^. Other pharmacological uses that had been studied as a preclinical phase^15^; depress the isolated perfused heart of the toad by direct action on the cardiac muscle. In the intact animal, an intravenous injection lowers the dog's systemic blood pressure, probably by direct action on the heart. There is no clear evidence as to the existence of any action on the blood vessels.

Nanostructured materials are essential building blocks for the fabrication of new devices. Nanotechnology is bringing up new ways to treat and prevent diseases^16^. Nanotechnology contributed to many fields such as imaging, sensing, targeting drug delivery systems, and synthesis of drugs like some anti-bacterial and anti-cancer drugs. It could be involved through synthesis design, production, characterization, production, and application by controlling the shape and size in the nanoscale range^17^.

Drug delivery, topicals, medicine, chemical sensing, data storage, cell biology, agriculture, cosmetics, textiles, the food industry, antioxidants, and antimicrobial agents are just a few fields where silver nanoparticles have made significant contributions^18^. Many sophisticated techniques were evolved for its synthesis as physical methods, including evaporation–condensation using a tube furnace at atmospheric pressure, laser ablation, and vacuum evaporation of metal^19, 20^. Many chemical approaches for its synthesis depend mainly on the reduction of silver by any reducing agents like sodium borohydride, ascorbic acid, and polyol process. Other methods rely on using organic solvents or stabilizing agents to prevent aggregation. Our approach depends only on using sodium borohydride as a reducing agent of silver in the ice bath for 30 s; then, the yellow solution of Ag NP's was formed without any heat or using any capping or stabilizing agents ^21^.

Ag NP's could be used as a fluorescence probe^22^, so many drugs could act as a quencher for its fluorescence according to Stern Volmer law^21^or absorptive probe and also could be quenched^23^ so their determination could be rapid, green, and rapid.

Spectrofluorometric assays for drugs could be used as an alternative to other sophisticated, time-consuming, expensive, and tedious analytical techniques. Our quenching-dependent fluorescence techniques are simple, novel, green, and rapid and could be used to quantify acemetacin in its pure form and pharmaceutical dosage forms. Both methods are valid according to ICH guidelines^24^.

Greening any analytical technique is challenging due to the sophisticated techniques or the significant usage of organic solvents. However, our methods would be flexible for greenness assessment as they are eco-friendly. Green analytical procedure index (GAPI), analytical eco scale, and green certificate are three tools used for measuring the green criteria of both methods.

The present work aimed to develop and validate a simple, sensitive and rapid spectrofluorimetric quenching-dependent method for determining ACM in its pure and pharmaceutical dosage form. The results obtained by the proposed method were in good agreement with those obtained by the comparison method.

## Methods

i) **Instrumentation**
- A Cary Eclipse fluorescence spectrophotometer (Agilent Technologies) was equipped with a xenon flash lamp. The slit width was 5 nm, with a 1.00 cm quartz cell.
- A Shimadzu UV–Visible double-beam spectrophotometer (Kyoto, Japan) UV-1601 PC with matched 1 cm quartz cells was utilized for the analysis.
- Absorption spectra of the studied drugs were recorded on a fast scan speed, setting slit width to be 1 nm
- The emission spectrum of acriflavine was quenched at 505 nm after excitation at 265 nm, using medium sensitivity and smoothing factor 19.
- The emission spectra of Ag NP's were quenched at 485 nm after excitation at 242 nm, using medium sensitivity and smoothing factor 19.
- The buffer solution pH was adjusted using a pH meter (Consort, NV P-901, Belgium).
- Model SS101H 230 for the Sonix IV (USA) A sonicator was employed.
- Transmission electron microscope (TEM), JEOL, JSM-2100(Tokyo, Japan). Sample was loaded on 200 carbon coated mesh and examined at 200KV.
ii) **Materials and reagents**
- Analytical-grade chemicals and solvents suitable for HPLC were utilized.
- Acemetacin was kindly provided by Multi Apex Pharma Pharmaceutical Industries- S.A.E, Badr City, Cairo, Egypt, with certified purity of 100.39%.
- Ost-map^®^ capsules contain 60 mg ACM (batch # RT05140620) produced by Apex for pharmaceutical industries- S.A.E, Badr City, Cairo, Egypt, and purchased from local pharmacies.
- Organic solvents like methanol, ethanol, acetonitrile and acetone were purchased from sigma Aldrich, Germany.
- Acriflavine was prepared at 8 × 10^−6^ M after being acquired from Sigma Aldrich in Germany.
- Silver nitrate and sodium borohydride were purchased from sigma Aldrich -Germany.
- The following surfactants were purchased from El-Nasr Pharmaceutical Chemicals Co. in Cairo, Egypt: tween 80, cetrimide, carboxy methyl cellulose (CMC), sodium dodecyl sulfate (SDS), and CMC.
- All three acids—acetic, boric, and phosphoric—and sodium hydroxide were bought from El-Nasr Pharmaceutical Chemicals Co. in Cairo, Egypt.
- Distilled water was used throughout the work.
iii) **Preparation of standard solutions, reagents, and buffer**
- The stock solution of ACM was prepared in methanol in concentration (100.0 µg/mL) and kept in aluminium foil as it is photosensitive.
- Briton–Robinson buffer (BRB) was prepared by mixing 0.04 M phosphoric acid, 0.04 M boric acid, and 0.04 M acetic acid and adjusting the pH using 0.2 M sodium hydroxide over the range of (pH 2–12).
- All surfactants were prepared in distilled water as 1% w/v solutions.
- Acriflavine solution was prepared as 8 × 10^−6^ M in distilled water. An aqueous solution of acriflavine (8 × 10^–4^ mol l^−1^; Sigma-Aldrich, Germany) consisting of 0.0208 g acriflavine in 100 ml distilled water was diluted to a concentration of 8 × 10^−6^ mol l^−1^
- Ag NP's were prepared (1.43 × 10^−4^ M) by drop-wise addition technique of 1 mM AgNO_3_ on 1 mM of sodium borohydride (NaBH_4_) in an ice bath with stirring from 30 s till 2 min maximum yellow solution of Ag NP's was prepared, stored at 5°C^21^and kept in aluminium foil. The prepared NPs were aged for 24 h in cool and dark place. They were mono-dispersible and stable for 3 weeks.
- Different concentrations of Ag NP's were prepared by changing other volumes of AgNO_3_ and NaBH_4_ in different ratios from (1:1) to (1:6).
iv) **Procedures**

A) Methods for calibration graphs.
- *Method I* Different aliquots of 100 µg/mL ACM were transferred in a 10-mL volumetric flask covering the concentration range in Table 1, adding 1 mL of BRB pH 6, then 1.5 mL of acriflavine reagent, completing the final volume by distilled water.
- *Method II* Different aliquots of 100 µg/mL ACM were transferred in a 10-mL volumetric flask, adding 1 mL of Ag NP's, completing the final volume by methanol, reaching the concentration range (1:16 µg/mL).
- Both methods were carried out at room temperature (immediately for Method I and after three minutes for Method II). The fluorescence intensity (FI) was measured at 505 nm in Method I following excitation at 265 nm, while the FI was measured at 485 nm following excitation at 242 nm in Method (II). A Blank experiment was performed, and the difference in fluorescence intensity between the blank and the calibration flasks (ΔF) was graphed against the corresponding drug concentrations in µg/mL. The regression equations were then derived in both methods.
B) Application to pharmaceutical dosage forms:
- Ten capsules of Ost-map^®^ were emptied and weighed, and then an equivalent amount of powder to 60 mg was considered, added to a 100 mL volumetric flask, and completed to the mark with methanol. The solution was sonicated for 30 min then filtered. Transferring a specific volume of the filtrate to a second 100-mL volumetric flask until the concentration reaches 100 µg/mL, the procedures under calibration curves were then performed, and the contents of the drug in capsules was calculated using the corresponding regression equation.

**Table 1:** Stern–Volmer parameters of the quenchers acriflavine (Method I) and AgNPs (Method II).

ACM (method I)				ACM (method II)		
Temperature (°K)	Stern–Volmer equation (Q, mol)	Correlation coefficient R^2^	K_SV_ (L.mol^−1^)	Stern–Volmer equation (Q, mol)	Correlation coefficient R^2^	K_SV_ (L.mol^−1^)
303	F_0_/F = 1.515 × 10^3^[Q] + 1.0495	0.996	1.515 × 10^3^	F_0_/F = 1.793 × 10^3^ [Q] + 1.1745	0.9982	1.793 × 10^3^
313	F_0_/F = 1.461 × 10^3^ [Q] + 1.0249	0.9945	1.461 × 10^3^	F_0_/F = 1.446 × 10^3^ [Q] + 1.126	0.9993	1.446 × 10^3^
323	F_0_/F = 1.313 × 10^3^ [Q] + 0.983	0.9996	1.313 × 10^3^	F_0_/F = 1.198 × 10^3^ [Q] + 1.0627	0.9983	1.198 × 10^3^

## Results and discussion

Quenching (intramolecular deactivation) transfers the non-radiative energy from the excited state to the other molecules^25^. Both acriflavine and Ag NP's were used as fluorescence probes so the studied drug could reach the optimum conditions because of the presence of the carboxylic group in the structure (Fig. 1).

**Figure 2:**
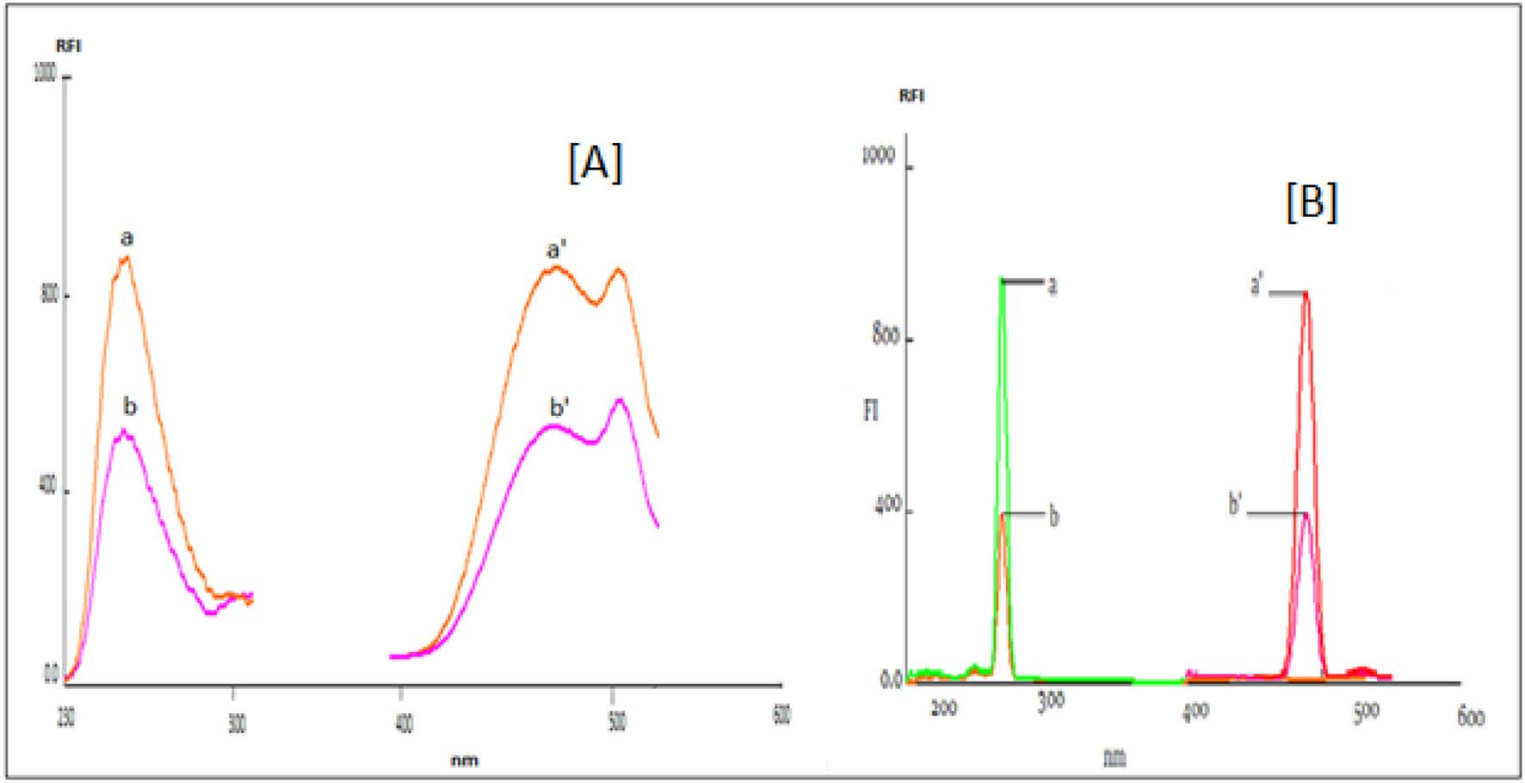
(**A**) a,a' are the excitation and emission spectra of 8 × 10^−6^ M acriflavine (1.5 mL). b,b' are the excitation and emission spectra of acriflavine in presence of 6 µg/mL ACM. (**B**) a,a' are the excitation and emission spectra of 1.43 × 10^−4^ M Ag NP's (1 mL). b,b' are the excitation and emission spectrum of Ag NP’s in presence of 6 µg/mL ACM.

**Figure 3:**
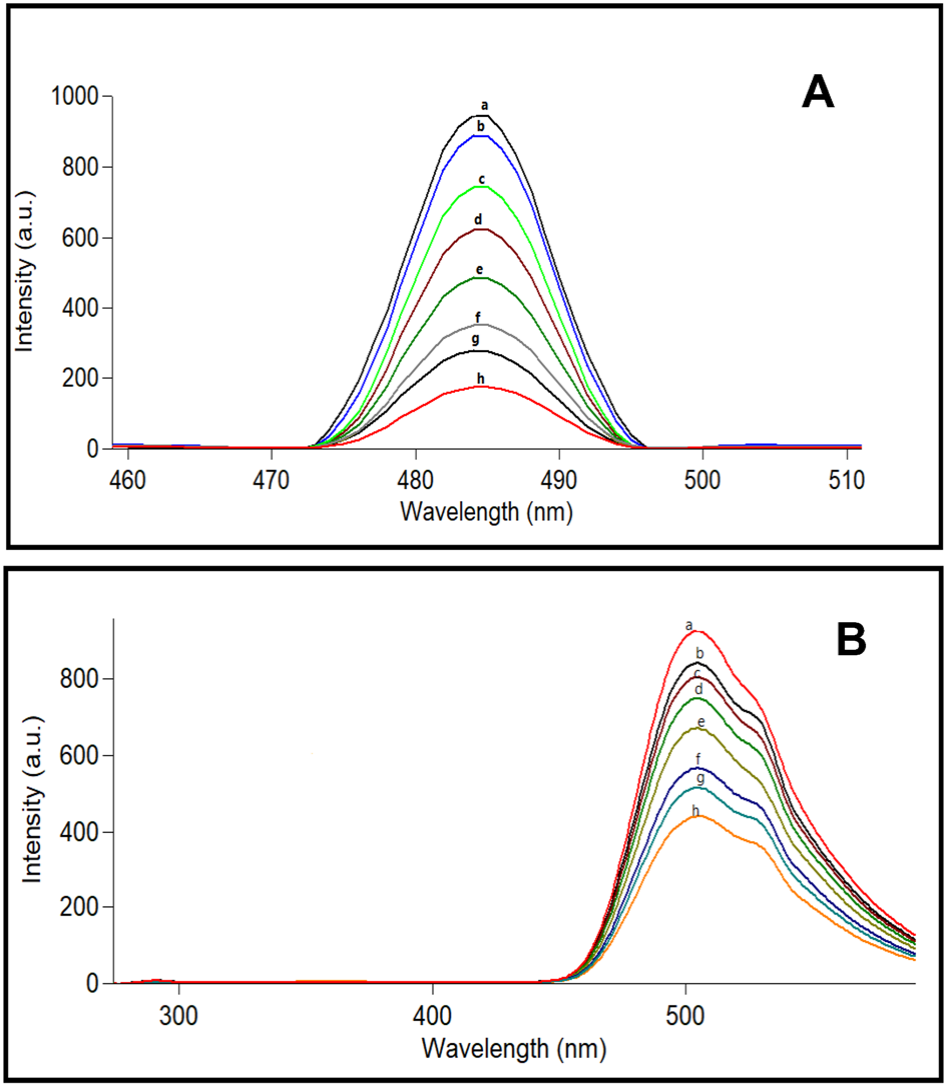
(**A**) The emissiion spectra of (a) 1.43 × 10^−4^ M of Ag NP's and from (b–h) are different concentrations of ACM (1–16 µg/mL) with Ag NP's (**B**) The emissiion spectra of (a) 8 × 10^−6^ M of Acriflavine and from (b–h) are different concentrations of ACM (2–20 µg/mL) with Ag NP's.

The process that causes a decrease in the fluorescence intensity of an analyte is known as fluorescence quenching^26^.Different molecular interactions involving energy transfer, ground–state complex formation, excited- state interactions, molecular measurements ,and collisional quenching can cause quenching process^26^. In this study, a sensitive, rapid, accurate, selective and ecofriendly spectrofluorimetric method was performed for the determination of ACM using acriflavine dye and Ag NP’s depending on the fact that acriflavine exhibits native fluorescence at 505 nm after excitation 265 nm and Ag NP’s exhibits native fluorescence at 485 nm after excitation at 242 nm. This fluorescence intensity decreases after the addition of ACM due to formation of non- fluorescent complex Figs. 2 and 3, respectively.

### Optimization conditions

The optimization of any method was studied by varying one parameter while fixing the other one using one concentration of ACM (6 µg/mL).

**Figure 4:**
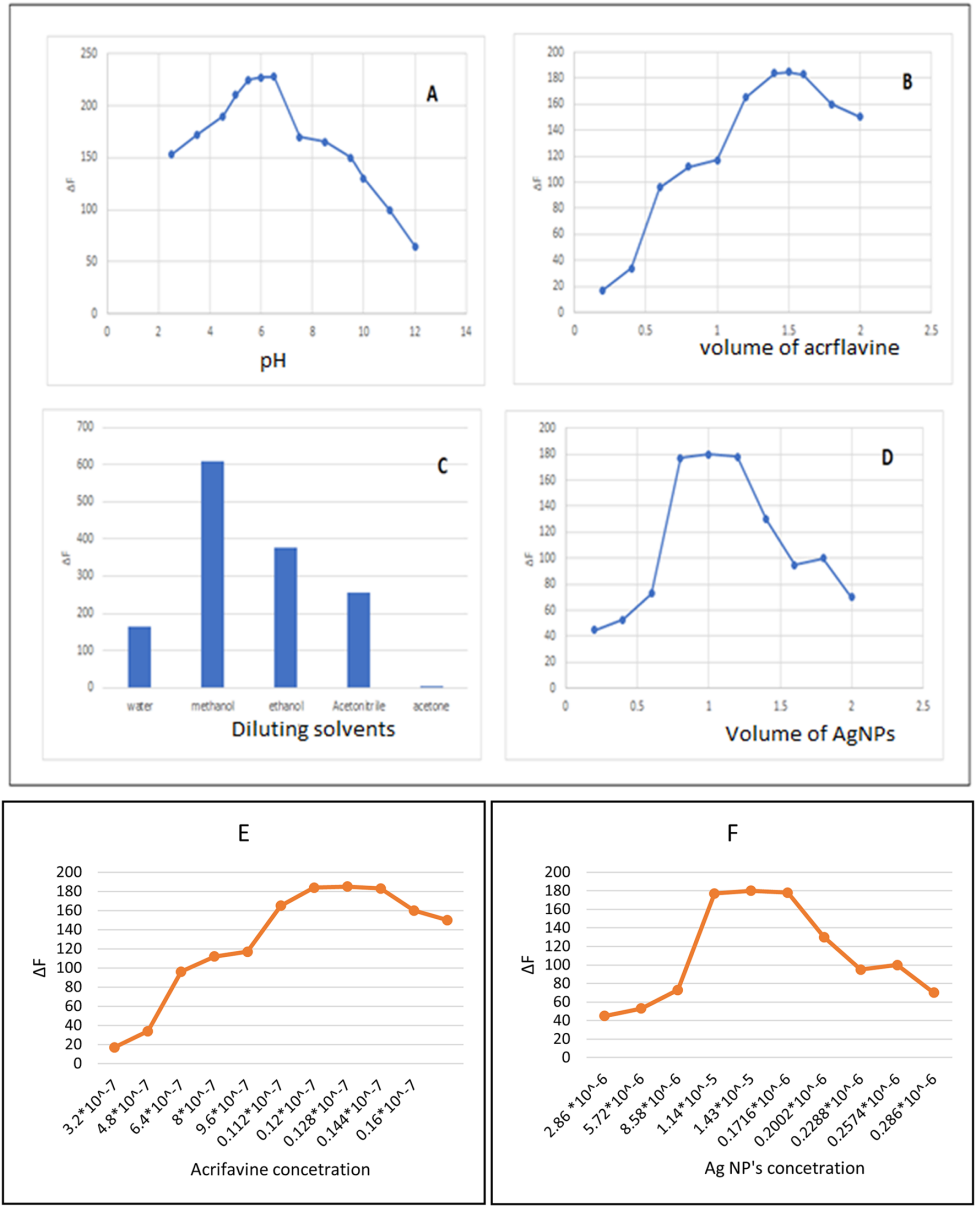
(**A**, **B**) The effect of pH and the volume of acriflavine, respectively in method I. (**C**, **D**) The effect of diluting solvents and the volume of silver, respectively in method II. (**E**, **F**) The effect of the concetrations of the reagents in method I and II, Respectively.

Different parameters were studied that affect both reactions to reach optimum conditions, like pH, buffer volume, reagent volume and concentration, and diluting solvents (Fig. 4).

#### Effect of diluting solvents.

Different diluting solvents were studied, like methanol, ethanol, acetonitrile, acetone, and distilled water, and the optimum one for Method I was water. In contrast, method II was methanol, as they gave a highly quantitative decrease in fluorescence intensity (∆F) of the reaction products.

#### Effect of pH

Briton Robinson buffer (BRB) was prepared to cover the range from pH 2–12, and the best pH that produces high (∆F) was pH 6 for the reaction product for Method I, while no buffer was added in Method II as there was no effect on the (∆F).

#### Effect of volume of buffer

Different volumes were studied starting from 0.2–2.0 mL, and the best volume that yields higher (∆F) in Method I was 1.0 mL as it produces high and reproducible (∆F).

#### Effect of organized media

Different surfactants were examined with 1% concentration. They include sodium dodecyl sulfate (SDS), β-cyclo dextrin (β-CD), tween-80, and carboxy methyl cellulose (CMC). Although SDS increased the (∆F) in Method I, the results are non-quantitative. No effect was observed in the case of using surfactant in Method II. So, no surfactant was incorporated into both methods. An aqueous buffered solution of ACM at pH 6 is the optimum condition for acriflavine quenching. The Ag NP's optimum quenching condition is only a methanolic solution of ACM.

#### Effect of volume and concentration of a reagent

The volume of acriflavine and Ag NP's were well evaluated by scanning different volumes, and the best ones for both reagents were 1.5 mL of 8 × 10^−6^ M stock solution (equivalent to a final concentration 0.12 × 10^−7^ M) and 1 mL of 1.43 × 10^−4^ M (equivalent to a final concentration 1.43 × 10^−5^ M), respectively, producing high (∆F).

#### Effect of contact time

The time of contact between 1.0 mL of Ag NP's or 1.5 mL of acriflavine and ACM solution was studied by monitoring the ∆F as a function of time using various time intervals, starting from 0 to 60 min. The interaction of [ACM-acriflavine] and [ACM-Ag NP's] was found to be completed immediately in Method I and after three minutes in method II, and was stable up to more than 60 min.

#### Spectral properties of Ag NP’s

- TEM images and UV spectral characteristics

Yellowish-dark spherical mono-dispersed Ag NP's were prepared in a ratio (1:6) of AgNO_3_ and NaBH_4_ with an average size of around ~ 10 nm, which was smaller than other methods using sodium citrate as a stabilizer. The narrow absorption peak of Ag NP's showed mono dispersity of Ag NP's at λ_max_ ~ 400 nm (Figure S1 A).

The UV spectrum of the prepared AgNPs was recorded and the obtained spectrum was found to have a narrow absorption peak near 400 nm (Figure. S1 B).

- Optimization of silver nanoparticles (concentration)

The fluorescence intensity of the system was greatly affected by the concentration of Ag NP's. Different concentrations of Ag NP's were prepared by adding varied volumes of sodium borohydride (NaBH_4_) to the same volume of silver nitrate (AgNO_3_) to form different ratios like (1 AgNO_3_:1,2,3,4,5,6,7,8,9 NaBH_4_) producing different concentrations of Ag NP's starts from 5 × 10^−4^ to 1 × 10^−4^ M/L, respectively. On the other hand, changing different volumes of AgNO_3_ molarity to the same volume of NaBH_4_ caused higher fluorescence intensity over the range that could not be determined. The optimum Ag NP's concentration was 1.43 × 10^−4^ M, while other concentrations are non-reproducible or have low fluorescence intensity resulting in decreasing ΔF or non-quenching action could be obtained (Figure. S2). Optimum Ag NP's could be measured at 485 nm after excitation at 242 nm at medium sensitivity, achieving a linear relationship between ΔF and the different concentrations of ACM.

- Fluorescence (FL) spectral characteristics for methods I and II

Static quenching resulted from the reaction between Ag NP's or acriflavine with ACM, decreasing their fluorescence intensity. This quenching was linear with the increase in ACM concentrations. The formation of the non-fluorescent complex results from the quenching of Ag NP's or acriflavine.

The Ag NP's emission spectra could be measured at 485 nm after excitation at 242 nm. Acriflavine emission spectra could be measured at 505 nm after excitation at 242 nm and 265 nm,

The final optimum calibration conditions in Method I are 1 mL of BRB pH 6, 1.5 mL of acriflavine (8 × 10^−6^ M), and diluting to 10 mL volumetric flask with distilled water while in method II after adding different aliquots of ACM, the addition of 1 mL of Ag NP's (1.43 × 10^−4^ M) and completing to the mark with methanol (Fig. 2).

#### Stoichiometry and mechanism of the reaction in methods I and II

Various ways were used for the determination of the stoichiometry of the reaction:

- Static or dynamic quenching depends on whether the complex formed between the reactants even in Method I or II on the ground or the excited state, respectively.
1. Job's continuous variation method
- *Method I:* ACM and acriflavine were prepared in the same concentration (8 × 10^–6^ M) to determine the reaction's stoichiometry.
- Different molar ratios of ACM and the reagent are complementary to each other to reach 1mL volume. as (0.1:0.9, 0.2:0.8, 0.3:0.7 and so on). The resulted (ΔF) was plotted against the volume fraction of the reagent, giving Job's continuous variation plot^27^.
- It was investigated to evaluate the reaction's stoichiometry between the fluorescent reagent (Acriflavine) and the quenching drug (ACM).
- The one carboxylic group in ACM interacted with the positively charged nitrogen atom in acriflavine, resulting in a 1:1 reaction molar ratio (Figure S3).
- The electrostatic interactions generated between them at an appropriate pH allowed the ion association complex to be established, which resulted in the formation of the non-fluorescent complex (Figure. S4).
2. Stern–Volmer equation was applied to examine the mechanism of quenching:
- Stern -Volmer law was applied to study the quenching mechanisms in both methods according to the following equation:
  $${\text{f}}_{{0}} {\text{/f = 1 + K}}\left[ {\text{Q}} \right]$$
  where: f_0_ and f are the fluorescence intensity of acriflavine or Ag NP’s alone and with the drug, respectively, K is the stern–Volmer (formation) constant and Q is ACM conc. Static quenching was the prominent as by increasing temperature, decreasing K hence decreasing the slope. This would be documented by plotting stern–Volmer plots for three different temperatures (303 K, 313 K and 323 K) as f_0_/f against the molar concentration of ACM. Stern–Volmer plots proved to be linear (Figure S5) in Table 1.
- Quenching due to inner filter effect (IFE) was detected by scanning the absorption spectra of different concentrations of the quencher (ACM) and the excitation and the emission fluorescence spectra of acriflavine and Ag NP's (Figure S6) showed some overlap, thus primary IFE might occur. The primary IFE was checked for ACM using this equation:
  $${\text{F}}_{{{\text{corr}}}} = {\text{F}}_{{{\text{obs}}}} \times {\text{antilog }}\left[ {{\text{A}}_{{{\text{ex}}}} + {\text{A}}_{{{\text{em}}}} /{2}} \right]$$
  where Aex and Aem are the absorbance values of the quencher at the excitation and emission wavelengths of the fluorophore, respectively, and Fobs is the observed fluorescence intensity, F_corr_ is the corrected fluorescence intensity after excluding IFE from F_obs_.
  Then, calculating %E, suppressed efficiency was calculated for both corrected and observed fluorescence according to this equation:
  $$\% {\text{E}} = \left[ {{1} - \left( {{\text{F}}/{\text{F}}_{0} } \right)} \right] \times {1}00$$
  Since the IFE accounts for about 18% and 20% of the total suppressed efficiency for ACM-Acriflavine and Ag NP’s, respectively, and the observed and corrected fluorescence of fluorescent probes is plotted against the quencher concentration (ACM) [M] (Figure S7), it can be concluded that the IFE plays a significant role in fluorescence quenching^28^.Therfore, the quenching mechanism was static with small inner filter effect.
3. Binding sites number, rate constant (K) and free energy change (DG°) calculation for the reaction of Ag NP's, Acriflavine and ACM^29, 30^.
  Different molecular interactions, including excited-state reactions, molecular rearrangements, ground-state complex formation and collisional quenching, may cause fluorescence quenching resulting from the complex construction. To assess the type of quenching that occurred, Stern–Volmer plots were constructed according to the following equation:
  $${\text{F}}_{{\text{o}}} /{\text{F}} = {1} + {\text{K}}_{{{\text{SV}}}} \left[ {\text{C}} \right],\;{\text{F}}_{{\text{o}}} /{\text{F}} = {1} + {\text{K}}_{{{\text{SV}}}} \left[ {\text{C}} \right],$$
  where *F* and *F*_o_ are the relative fluorescence intensities of acriflavine and Ag NP's with and without the drug, respectively, *K*_SV_ is the Stern–Volmer constant and [C] is the molar concentration of the drug.
- The binding constant K and the binding sites (n) between ACM and Ag NP's and acriflavine was calculated by utilizing this eqn
  $${\text{log }}({\text{F}}{-}{\text{F}}_{{\text{o}}} )/{\text{F}}) = {\text{logk}} + {\text{nlog}}[{\text{D}}]$$
  where; [D] is the molar concentration of ACM.
- Estimating the number of binding sites (n) between ACM, Ag NP's and Acriflavine was approximately one, as represented by the slope value (1.052) and (1.015). The binding constant (K were 7.9 × 10^3^ and 2.6 × 10^3^, respectively as the intercept.
  (log K) were 3.9 and 3.42, respectively.
  **Gibb's free energy (DG°)** was calculated by utilizing K value through eqn.
- Gibb's free energy (D*G*°, kJ mol^−1^)
  $$= - RT{\text{ln}}K = - {22631}.{62} {\text{kJ}} {\text{mol}}^{{ - {1}}} \;\;\left( {\text{method I}} \right)$$
  $$= - RT{\text{ln}}K = - {19822}.{85} {\text{kJ}} {\text{mol}}^{{ - {1}}} \;\; \left( {\text{method II}} \right)$$
  where; R is the universal gas constant (8.314 J K^−1^ mol^−1^), T is the temperature in Kelvin, A negative value for DG indicates a spontaneous process.
4. The bimolecular quenching constants (*K*_q_) were calculated to indicate the fluorescence efficiency according to the following Eq. ^30^
  $${\text{k}}_{{\text{q}}} \, = \,{\text{K}}_{{{\text{SV}}}} /\tau_{{\text{o}}}$$
  The fluorescence lifetime of acriflavine and AgNPs are 5 × 10^−9^ s, 1.86 × 10^−9^ s so K_q_ was found to range from 3.03 to 9.65 × 10^11^ l mol^−1^ s^−1^.

Method (I):

$$\begin{aligned} {\text{Kq}} = & {\text{k}}_{{\text{q}}} \, = \,{\text{K}}_{{{\text{SV}}}} /\tau_{{\text{o}}} \\ = & { 1}.{\text{515 x 1}}0^{{3}} /{\text{ 5 x 1}}0^{{ - {9}}} \\ = & { 3}.0{\text{3 x 1}}0^{{{11}}} {\text{l mol}}^{{ - {1}}} {\text{s}}^{{ - {1}}} . \\ \end{aligned}$$

Method (II):

$$\begin{aligned} {\text{Kq}} = & {\text{k}}_{{\text{q}}} \, = \,{\text{K}}_{{{\text{SV}}}} /\tau_{{\text{o}}} \\ = & {1}.{793} \times {1}0^{{3}} /{1}.{86} \times {1}0^{{ - {9}}} \\ = & {9}.{65} \times {1}0^{{{11}}} {\text{l}}.{\text{ mol}}^{{ - {1}}} .{\text{ S}}^{{ - {1}}} \\ \end{aligned}$$

### Validation criteria

The proposed methods were examined regarding the validation parameters as linearity, range, accuracy, precision, detection, and quantitation limits according to ICH guidelines^24^. An excellent linear relationship exists between the ∆F and drug concentration in ranges 2.0–20.0 µg/mL and 1.0–16.0 µg/mL for methods I and II, respectively (Table 2) (Figure S8).

**Table 2:** Analytical performance data for the determination of ACM by the proposed methods.

Parameter	Method (I) Acriflavine	Method (II) AgNPs
Drug	ACM	ACM
Wavelength (nm)	265 nm/505 nm	242 nm/485 nm
Linearity range (µg/mL)	2–20	1–16
Intercept (a)	85.16	− 3.19
Slope (b)	20.47	47.83
Correlation coefficient (r)	0.9998	0.9998
SD of residuals (Sy/x)	3.11	5.80
SD of intercept (Sa)	2.42	4.59
SD of slope (Sb)	0.19	0.43
Percentage relative standard deviation, % RSD	0.96	0.87
Percentage relative error, % error	0.39	0.36
Limits of detection, LOD (µg/mL)	0.39	0.32
Limits of quantitation, LOQ (µg/mL)	1.18	0.96

Accuracy was checked by calculating the percentage recovery of the pure drug with the comparison method^4^, and data are summarized in Table 3. The comparison method^4^ was based on zero-order UV spectrophotometry, where the absorbance of ACM methanolic solution was measured at 280 nm. The statistical analysis results showed no significant difference between the suggested methods and the reported Method, proved by the student's t-test and variance ratio *F*-test^31^ on the levels of accuracy and precision.

**Table 3:** Assay results for the determination of the studied drugs in pure form by the proposed and comparison methods.

Parameters									
ACM (µg/mL)	Method I (Acriflavine)			Method II (AgNPs)			Comparison method [4]		
	Amount taken µg/mL	Amount found µg/mL	% Found^a^	Amount taken µg/mL	Amount found µg/mL	% Found^a^	Amount taken µg/mL	Amount found µg/mL	% Found^a^
	2.00	1.983	99.15	1.00	1.008	100.80	10.00	9.911	99.11
	4.00	3.951	98.78	3.00	3.014	100.47	15.00	15.252	101.68
	8.00	8.035	100.44	9.00	9.056	100.62	20.00	19.869	99.35
	12.00	12.153	101.28	12.00	11.897	99.14			
	16.00	16.052	100.33	14.00	13.862	99.01			
	20.00	19.857	99.29	16.00	16.163	101.02			
Mean			99.88			100.17			99.88
± SD			0.96			0.87			1.12
% RSD			0.96			0.87			1.12
% Error			0.39			0.36			0.49
*t* test			0.21			0.17			
*F* value			2.2			2.63			

NB ^a^Mean of three determinations.

The tabulated *t* and *F* values at 2.77 and 19 at *P* = 0.05, respectively [30].

The low percentage of relative standard deviations and relative errors proved sufficient precision through three different concentrations at three consecutive times in a day (intraday precision) or three consecutive times in three days (interday precision) (Table 4).

**Table 4:** Precision data for the estimation of studied drugs by the proposed methods.

Parameters drug (ACM) (µg/mL)		Method (I) (Acriflavine)			Method (II) (AgNPs)		
		2.00	12.00	16.00	12.00	14.00	16.00
Intra-day	Mean	100.1	99.88	99.93	99.99	99.99	99.88
	± SD	1.25	1.82	0.65	0.79	0.39	0.89
	% RSD	1.25	1.82	0.65	0.79	0.39	0.89
	% Error	0.72	1.05	0.38	0.45	0.22	0.52
Inter-day	Mean	100.31	100.45	99.66	100.31	99.99	99.99
	± SD	1.39	1.45	1.20	1.39	1.62	1.20
	% RSD	1.39	1.45	1.21	1.39	1.62	1.20
	% Error	0.80	0.84	0.69	0.80	0.94	0.69

N. B. Each result is the average of three separate determinations.

Robustness was investigated by considering minor variations in the two methods; in Method I: the reagent volume, the buffer volume, and pH, and in Method II; the reagent volume as indicated in (Table 5).

**Table 5:** Robustness of both methods.

Parameters	Mean	S. D	%RSD	%Error
Method I				
pH 6 ± 0.2 (5.8:6.2)	100	± 0.13	0.13	0.075
1 mL BRB pH 6 ± 0.2 (0.8:1 mL)	100	± 0.43	0.43	0.25
1.5 mL Acriflavine ± 0.2 (1.3:1.7 mL)	99.99	± 0.46	0.46	0.27
Method II				
1 mL AgNPs ± 0.2 (0.8:1 mL)	99.96	± 0.81	0.81	0.466

N. B. Each result is the average of three separate determinations.

Detection and quantitation limits for both methods, respectively, are calculated by applying ICH mathematical Eqs. ^25^ and were found to be 0.39, 0.32, and 1.18, 0.96, respectively.

Both methods were in good agreement with the comparison method^4^, and they could easily be applied to the pharmaceutical preparations with good accuracy and precision with no interference from non-pharmaceutical ingredients, which described the selectivity of the methods.

### Application to the pharmaceutical preparation

The investigated pharmaceutical preparation: Ost-map^®^ contains 60 mg of ACM, was analyzed using the two methods. Acceptable accuracy and precision values were obtained, and no significant difference with the published method was noticed^4^ after calculating the student's *t*-test and *F*-value^31^ as indicated in (Table 6) and Figure (S.9).

**Table 6:** Determination of ACM in pharmaceutical preparations using the proposed methods.

Parameters	Proposed method			Comparison method		
	Amount taken	Amount found	Percentage	Amount taken	Amount found	Percentage
Method(I)	(µg/mL)	(µg/mL)	Found ^a^	(µg/mL)	(µg/mL)	Found ^a^
Ost-map^®^	8.00	7.922	99.03	10	9.911	99.11
Capsules	12.00	12.194	101.62	15	15.252	101.68
ACM (60 mg)	16.00	15.889	99.31	20	19.869	99.35
Mean			99.99			99.88
± SD			1.42			1.12
% RSD			1.42			1.12
% Error			0.82			0.49
*t* test				0.05		
*F* value				1.00		
Method (II)						
Ost-map^®^	12.00	11.88	99.03			
capsules	14.00	14.22	101.62			
ACM (60 mg)	16.00	15.88	99.33			
Mean			99.99			
± SD			1.42			
% RSD			1.42			
% Error			0.82			
*t* test				0.046		
*F* value				1.00		

N.B. ^a^Mean of three determinations.

The tabulated *t* and *F* values at 2.77 and 19 at *P* = 0.05, respectively [30].

### Greenness property

Three methods were followed for the assessment of greenness. The first one is the green analytical procedure index (GAPI)^32^. It deals with the sample from its collection to waste treatment. It consists of 15 items through five pentagrams. Each item was also assessed through three colours: green (higher green), yellow (medium green), and red (lower green under normal circumstances, ACM and Ag NP's must be stored in aluminium foil and a refrigerator, which is why the 4th parameter was yellow-shaded in both ways. The fifth parameter was coloured yellow since both procedures involved sample preparation and filtration. Field No. 15 in all techniques had red colouring because there was no waste treatment, and the amount of waste was between 1 and 10 mL; thus, it was tinted yellow. Another approach is the analytical eco scale, which works by keeping track of the penalties during the entire analytical process and then deducting them from 100^33^. In both methods, the scores were 90 and 93 owing to the usage of almost non-hazardous chemicals according to "The Globally Harmonized System of Classification and Labelling of Chemicals" (GHS) and the safety label data sheet for each chemical. The last method is called the green certificate^34^. It is classified into colours from green colour, lower eco-impact to red one, higher negative eco-impact, and letters from A (the greenest) to G (the least green). Method, I categorized A for reagent consumption and waste generation, while the other process classified B for reagent consumption and A for waste generation. so our methods remain in good line with the environment (Table 7).

**Table 7:** Results for the evaluation of the developed methods by the three green chemistry tools (Method I &II).

*1-Green analytical procedure index (GAPI)*			
*Method I*	*Method II*		
	
*2-Analytical eco scale score*			
*Method I*			
Reagents/instruments			
Reagent, volume (mL)	No of Pictograms	Word sign	Penalty points
BR buffer, 1 mL	1	Danger	2
Acriflavine, 1.5 mL	2	Warning	2
Water		0	
Item			Penalty points
Spectrofluorometer	< 0.1 k w h per sample		0
Waste	No treatment		3
Occupational hazards	Analytical process hermitization		0
Total penalty points			$\sum 7$
Analytical eco scale score			100 − 7 = 93
*Method II*			
Reagents/instruments			
Reagent, volume (mL)	No of Pictograms	Word sign	Penalty points
AgNPs,1 mL	1	warning	1
Methanol, 9 mL	3	Danger	6
Item			Penalty points
Spectrofluorometer	< 0.1 k w h per sample		0
Waste	No treatment		3
Occupational hazards	Analytical process hermitization		0
Total penalty points			$\sum {10}$
Analytical eco scale score			100 − 10 = 90
*Green certificate*			

## Conclusion

Novel spectrofluorometric methods depending on acriflavine or Ag NP's fluorescence quenching mechanisms were applied to determine ACM with the merits of effectiveness, simplicity, and selectivity. Both methods could be used for bulk powder and pharmaceutical preparations with good accuracy and precision. According to the international conference for harmonization (ICH) guidelines, the methods proved valid. Different green assessment tools checked the greenness of the two methods. Both methods could be used as an alternative, sophisticated methods in quality control units or for medical purposes.

## Supplementary Information

Supplementary Information 1.

Supplementary Information 2.

Supplementary Information 3.

## Data Availability

All data generated or analyzed during this study are included in this published article [and its supplementary information files].
