# Supplementary material for: Investigation of eco-friendly fluorescence quenching probes for assessment of acemetacin using silver nanoparticles and acriflavine reagent
Source: Sci Rep. 2023 Mar 14;13:4237. doi: 10.1038/s41598-023-31106-9 (PMC10014932; doi:10.1038/s41598-023-31106-9)
Supplement: Supplementary file 3 — Supplementary Information 3. [file 41598_2023_31106_MOESM3_ESM.docx]

**Investigation of eco-friendly fluorescence quenching probes for assessment of acemetacin using silver nanoparticles and acriflavine reagent**

**Rana Ghonim ^a, b*^, Mohamed I. El-Awady ^a, b^, Manar M. Tolba ^a^, Fawzia Ibrahim ^a^**

1. *Department of Pharmaceutical Analytical Chemistry, Faculty of Pharmacy, Mansoura University, Mansoura 35516, Egypt.*
2. *Department of Pharmaceutical Chemistry, Faculty of Pharmacy, Delta University for Science and Technology, International Coastal Road, Gamasa 11152, Egypt.*

**Supplementary Information:**

**Fig. S1:** TEM images and UV spectral characteristics of Ag NP’s:

A. TEM images of 1.43 ×10^-4^ M Ag NP’s with the capsule analyzed in (d).

B. Absorbance spectrum of the prepared AgNPs (1.43 × 10^−4^ M).

**Fig. S2:** Different concentrations of Ag NP’s prepared by different ratios of molarity of each AgNO_3_:NaBH_4_ (1:1,2,3,4,5,6,10), respectively:

**a** is 3.3 × 10^-4^ M, **b** is 1.43× 10^-4^ M, **c** is 1 × 10^-4^ M, **d** is 2 × 10^-4^ M, **e** is 2.5 × 10^-4^ M, **f** is 1.66 × 10^-4^ M, **g** is 0.91 × 10^-4^ M [Ag NP’s].

**Fig. S3:** Stoichiometry of the reaction explained by Job’s method in method I.

**Fig. S4:** Mechanism of ion- association complex between ACM and acriflavine.

**Fig. S5:** A, B are stern-Volmer plots for quenching florescence at 303 K,313 K and 323 K for and acriflavine (A) and Ag NP’s (B) , respectively.

**Fig. S6:** a is the UV absorption spectra of ACM

b is the excitation and the emission spectra of Ag NP’s

c is the excitation and the emission spectra of acriflavine

**Fig. S7:** A, B are Suppressed efficiency of observed and corrected fluorescence of Ag NP’s (A) and acrifavine (B), respectively. after addition of different concentrations of ACM.

**Fig. S8:** The standard calibration curve for method I (A) and method (II) (B), illustrating the slope and the intercept

**Fig. S9:** (A): The emission spectra of (a) acriflavine and different concentrations of OST-Map^®^ capsules (b-d) (8-12-16 µg/mL).

(B): The emission spectra of (b) Ag NP’s and different concentrations of OST-Map^®^ capsules (b-d) (3-9-14 µg/mL).


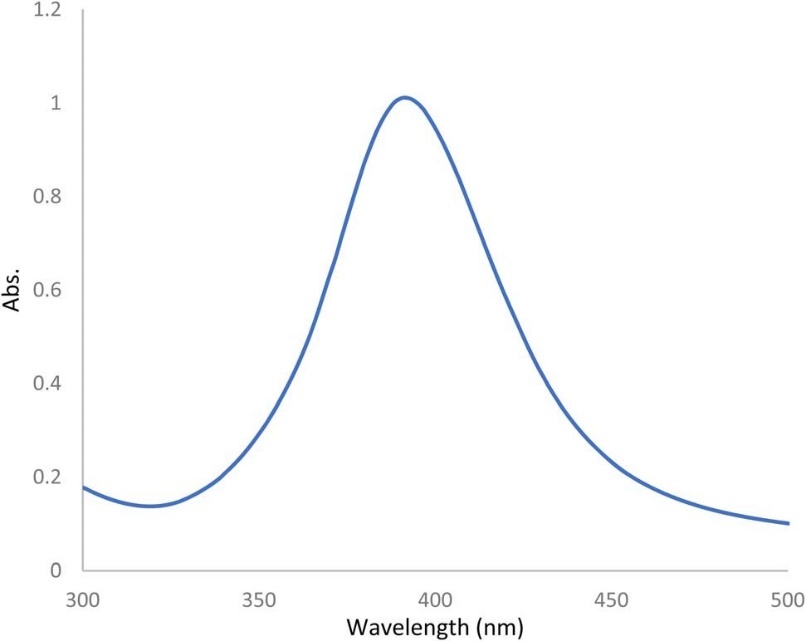

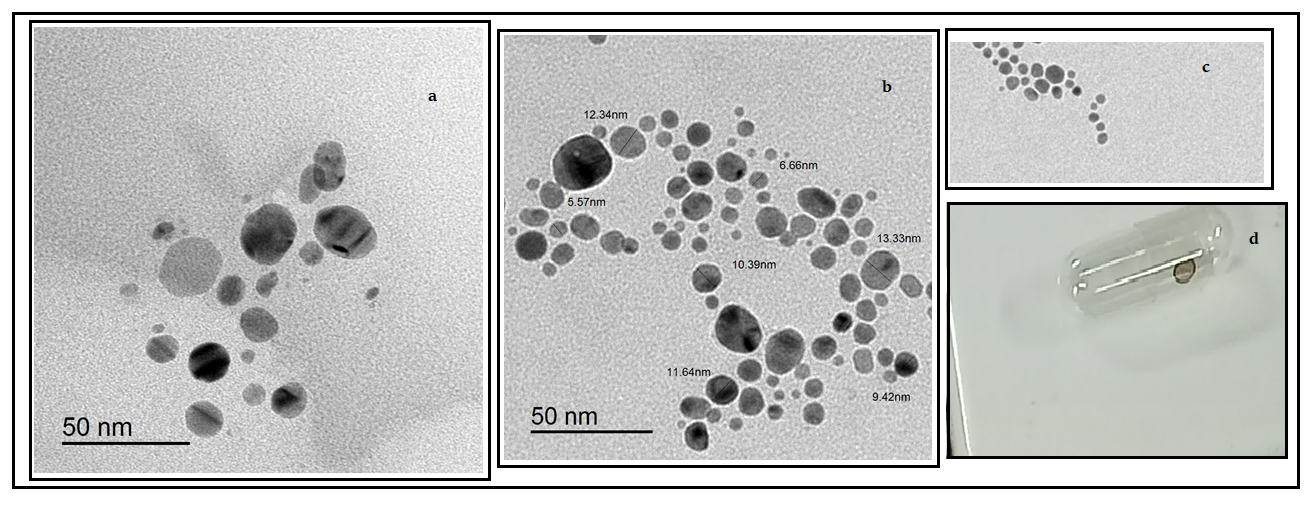


**A**

**B**

**Fig. S1:** TEM images and UV spectral characteristics of Ag NP’s:

A. TEM images of 1.43 ×10^-4^ M Ag NP’s with the capsule analyzed in (d).

B. Absorbance spectrum of the prepared AgNPs (1.43 × 10^−4^ M).


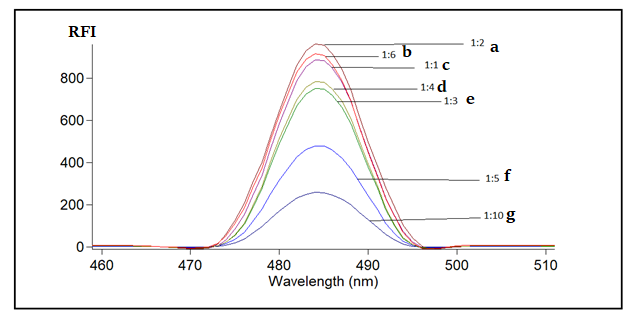


Fig.S2:

Different concentrations of Ag NP’s prepared by different ratios of different molarity of each of AgNO_3_:NaBH_4_ (1:1,2,3,4,5,6,10), respectively:

**a** is 3.3 × 10^-4^ M, **b** is 1.43× 10^-4^ M, **c** is 1 × 10^-4^ M, **d** is 2 × 10^-4^ M, **e** is 2.5 × 10^-4^ M, **f** is 1.66 × 10^-4^ M, **g** is 0.91 × 10^-4^ M [Ag NP’s].


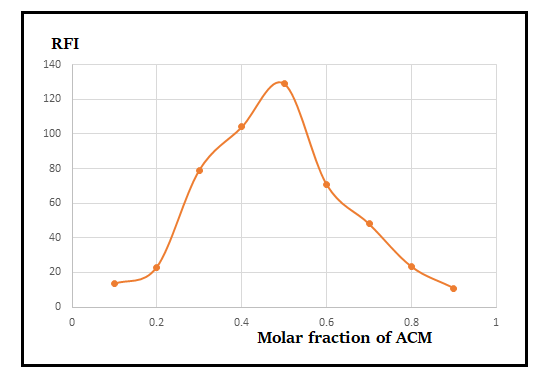


Fig. S3:

Stoichiometry of the reaction explained by Job’s method in method I.


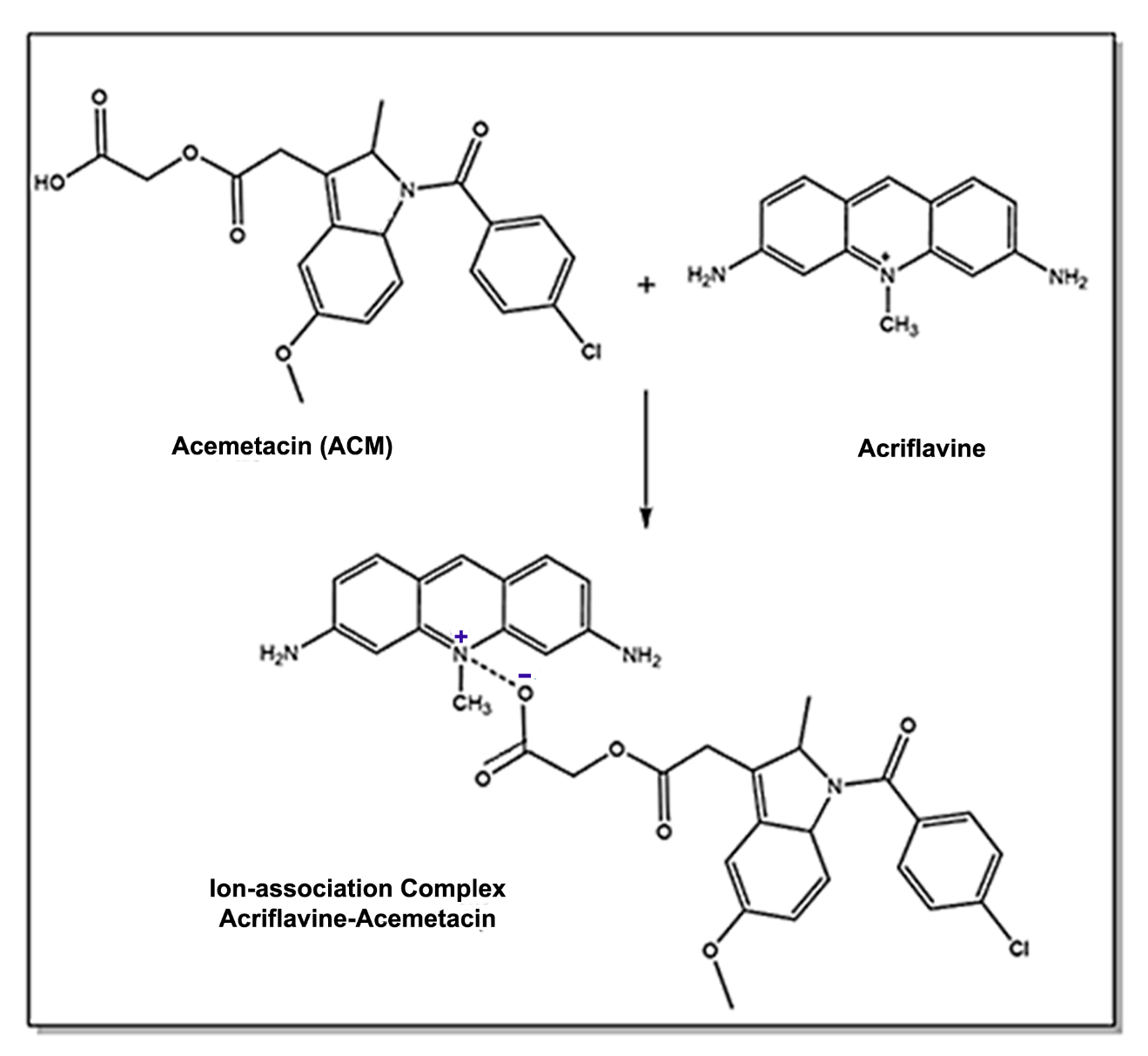


Fig.S4: Mechanism of ion- association complex between ACM and acriflavine.

Fig.S5: A, B are stern-Volmer plots for quenching florescence at 303 K,313 K and 323 K for acriflavine (A) and Ag NP’s (B), respectively.


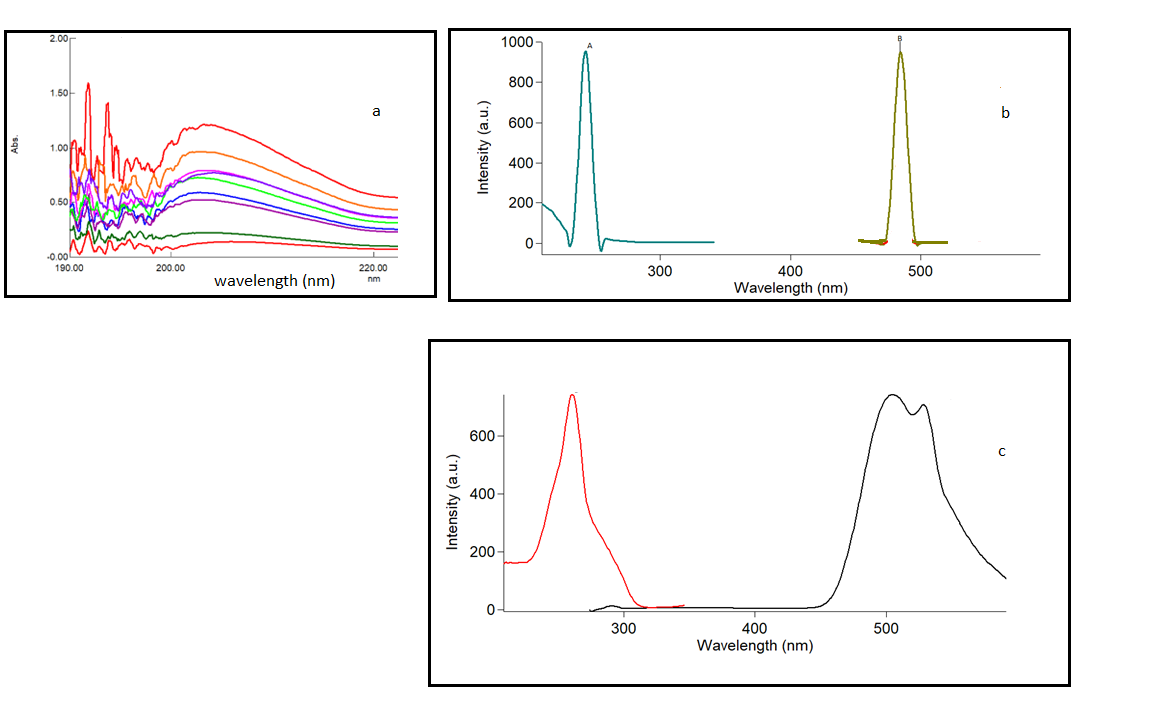


Fig.S6: a is the UV absorption spectra of ACM

b is the excitation and the emission spectra of Ag NP’s

c is the excitation and the emission spectra of acriflavine


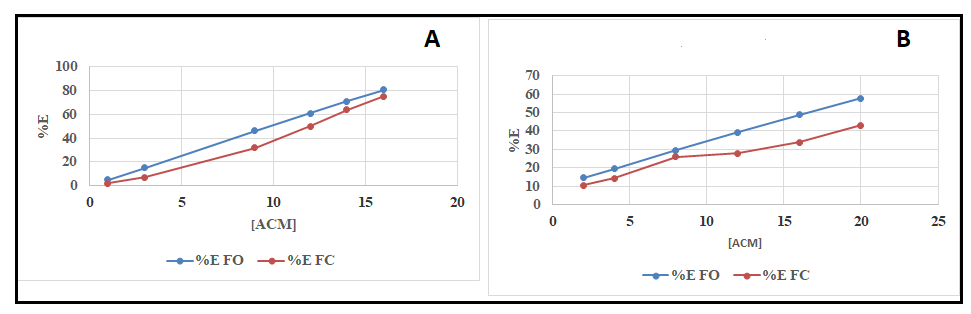


Fig.S7: A, B are Suppressed efficiency of observed and corrected fluorescence of Ag NP’s (A) and acrifavine (B), respectively. after addition of different concentrations of ACM.

Fig.(S8): The standard calibration curve for method I (A) and method (II) (B), illustrating the slope and the intercept


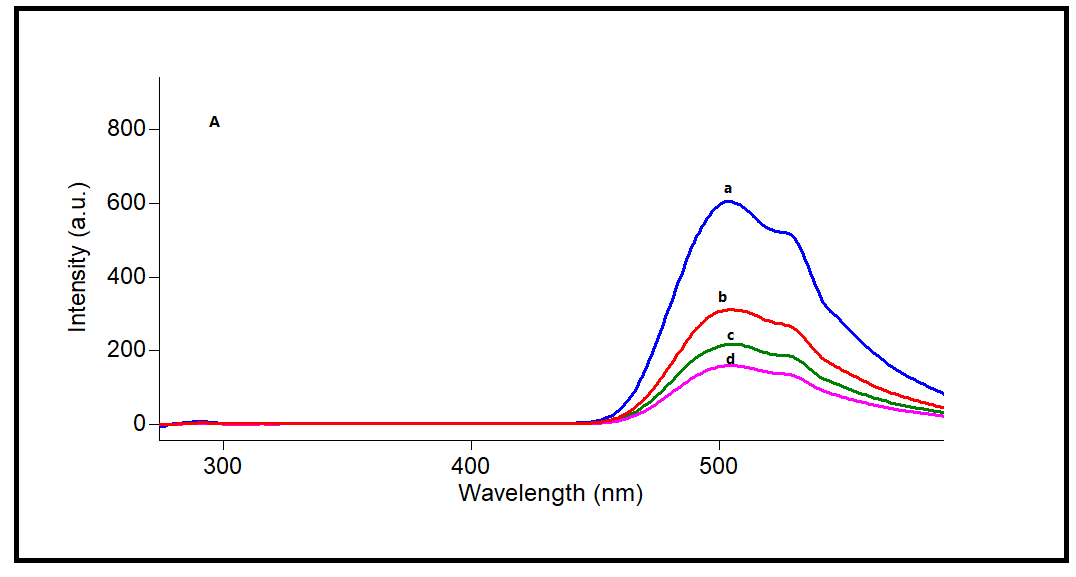


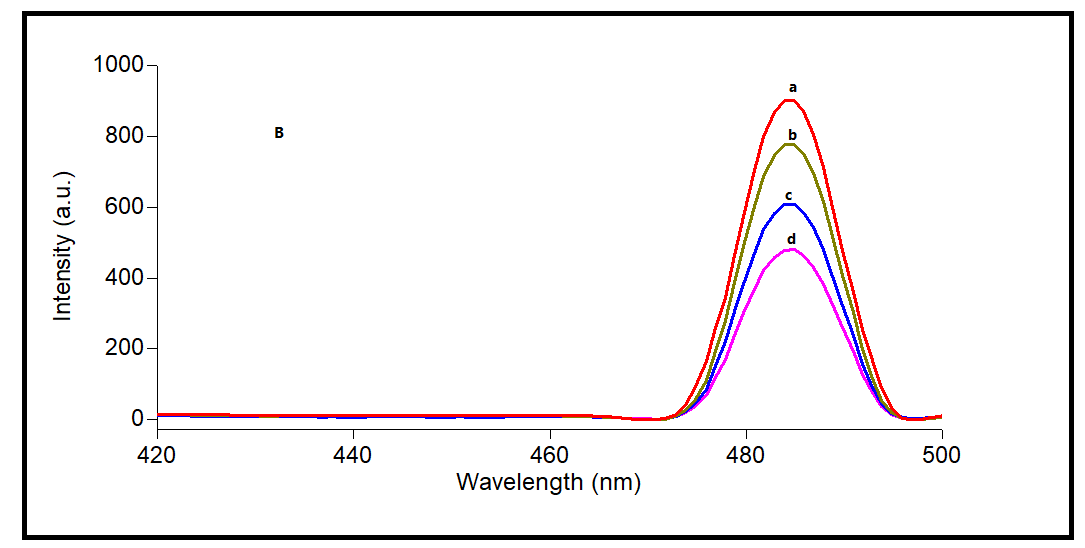


Fig. (S9):

(A): The emission spectra of (a) acriflavine and different concentrations of OST-Map^®^ capsules (b-d) (8-12-16 µg/mL).

(B): The emission spectra of (b) Ag NP’s and different concentrations of OST-Map^®^ capsules (b-d) (3-9-14 µg/mL).
